# mRNA expression and hypermethylation of tumor suppressor genes apoptosis protease activating factor-1 and death-associated protein kinase in oral squamous cell carcinoma

**DOI:** 10.3892/ol.2013.1353

**Published:** 2013-05-17

**Authors:** CHUNYAN LI, LIN WANG, JING SU, RUHUI ZHANG, LI FU, YANMIN ZHOU

**Affiliations:** 1 Departments of Implant Center, Jilin University, Changchun, Jilin 130011, P.R. China;; 2 VIP Integration, School of Dentistry, Jilin University, Changchun, Jilin 130011, P.R. China;; 3 Department of Pathophysiology, School of Basic Medical Sciences, Jilin University, Changchun, Jilin 130011, P.R. China;; 4 Department of Oral and Maxillofacial Surgery, School of Dentistry, Jilin University, Changchun, Jilin 130011, P.R. China

**Keywords:** methylation, apoptosis protease activating factor-1, death-associated protein kinase, oral squamous cell carcinoma, 5-aza-2′-deoxycytidine

## Abstract

Apoptosis protease activating factor-1 (Apaf-1) and death-associated protein kinase (DAPK) are p53 pathway-related genes that play significant roles in the activation of caspases, which are involved in mitochondrial-mediated apoptosis. The present study aimed to confirm the role of hyper-methylation of the Apaf-1 and DAPK gene promoter regions in oral squamous cell carcinoma (OSCC) and the effect of the demethylation drug, 5-aza-2′-deoxycytidine (DAC). mRNA from 53 OSCC samples, 23 normal oral mucosa samples and Tca8113 human tongue carcinoma cell lines was detected using semi-quantitative reverse transcription-polymerase chain reaction (RT-PCR). The DNA from each sample was analyzed using methylation-specific PCR (MSP). The Tca8113 cells were demethylated using DAC and the demethylation and re-expression of Apaf-1 and DAPK were analyzed. The Apaf-1 and DAPK mRNA expression index was decreased in 51 (96.23%) and 50 (94.34%) cases, respectively, in the tumor tissues. Hypermethylation of the Apaf-1 and DAPK promoter regions was detected in 46 (86.79%) and 38 (71.69%) cases, respectively. Promoter hypermethylation of the two genes correlated with a decreased mRNA expression in the tumor tissues. Subsequent to being treated with DAC, Apaf-1 and DAPK were demethylated and re-expressed in the Tca8113 cells. Apaf-1 and DAPK promoter hypermethylation may be associated with low gene expression in OSCC. Furthermore, a loss of Apaf-1 and DAPK expression may recover following demethylation. The data provide evidence that methylation exists in OSCC and may play a role in the development of this disease.

## Introduction

Oral squamous cell carcinoma (OSCC) is one of the most common malignant tumors of the oral and maxillofacial region. The occurrence and development of OSCC are multi-stage processes involving a variety of changes at the gene level, including the activation of oncogenes and the inactivation of tumor suppressor genes (1). Apoptosis is a mechanism that is responsible for the physiological deletion of cells and appears to be intrinsically programmed in certain physiological or pathological conditions. The inactivation of apoptosis-related tumor suppressor genes may lead to abnormal cell proliferation and eventually to tumorigenesis.

Apoptosis protease activating factor-1 (Apaf-1) and death-associated protein kinase (DAPK) are apoptosis-related tumor suppressor genes that have generated interest due to their downregulated expression in various tumors. Studies have shown that their reduced expression is associated with promoter hypermethylation (2–5). DAPK is able to activate the p19ARF/p53-dependent apoptotic pathway through phosphorylation of p19ARF (6). p53 then triggers the mitochondrial apoptotic pathway, leading to DNA fragmentation and apoptosis by inducing the expression of certain specific apoptotic genes, including Bax, PUMA, Noxa and Apaf-1. The significance of p53 in initiating the early stages of apoptosis has been widely confirmed (7). Therefore, studies with regard to the expression of Apaf-1 and DAPK in OSCC may contribute to exploring the anti-apoptotic pathway of tumor cells and may also provide guidance for future treatment.

The expression and methylation levels of Apaf-1 and DAPK in OSCC tissues or cells have rarely been reported. The present study detected the expression and methylation of Apaf-1 and DAPK in OSCC tissues and Tca8113 tongue squamous cell carcinoma cell lines. Demethylation was also observed in the transcriptional regulation of Apaf-1 and DAPK in the Tca8113 cell line.

## Materials and methods

### Patients, diagnosis and samples

A total of 33 male and 20 female patients with OSCC, with a median age of 55 years (range, 40–72 years), were selected for the study. A control group comprising 23 cases of normal oral mucosa was used. No patients were administered antitumoral treatment prior to the tumor samples being taken. The diagnosis of OSCC was based on standard criteria. A total of 19 patients (35.8%) were classified with stage I tumors, 25 (47.2%) with stage II and 9 (17%) with stage III. All the samples were obtained using aseptic techniques in the operating theatre and placed into liquid nitrogen immediately. No patients were administered chemotherapy or radiotherapy prior to surgery. All the samples were obtained according to the agreement of the ethics committee of the Medical Department of Jilin University (Changchun, Jilin, China).

### Reverse transcription-polymerase chain reaction (RT-PCR)

Total RNA was extracted from 53 OSCC samples, 23 normal oral mucosa samples and from the Tca8113 cell line using TRIzol (Invitrogen, Carlsbad, CA, USA), according to the manufacturer’s instructions, and stored at −80°C. All RNA samples with an A260/280 ratio >1.9 were selected for the RT procedure using a Quantscript RT kit (Tiangen, Beijing, China). cDNA amplification and the detection of specific products were performed using PCR. Apaf-1 and DAPK expression was determined on the basis of the endogenous control, β-actin. The specific primer sequences are given in Table I. PCR was performed in a thermal cycler using the following cycling conditions: 94°C for 5 min, 30 cycles at 94°C for 30 sec, 55°C for 30 sec, 72°C for 30 sec and a final extension of 10 min at 72°C. The PCR mixture contained 2 *μ*l cDNA, 1 *μ*l of each primer (10 *μ*M), 12.5 *μ*l 2X Taq MasterMix in a final volume of 25 *μ*l. The 10 *μ*l PCR products were then loaded onto 1.5% (m/v) agarose gel, electrophoresed and visualized under ultraviolet light subsequent to being stained with ethidium bromide.

### Methylation-specific PCR (MSP)

Genomic DNA was extracted from 53 OSCC samples, 23 normal oral mucosa samples and from the Tca8113 cell line using the TIANcombi DNA Lyse&Amp PCR kit (Tiangen). MSP was performed on the Apaf-1 and DAPK promoter regions. The DNA was modified and purified using the EpiTect Bisulfite kit (Qiagen, Hilden, Germany). The primer sequences that were used to detect the methylated and unmethylated promoters in the Apaf-1 and DAPK genes are shown in Table II. MSP was performed in a thermal cycler using the following cycling profile: Apaf-1, 94°C for 5 min, 39 cycles at 94°C for 30 sec, 61°C for 90 sec, 72°C for 60 sec and a final extension of 5 min at 72°C; DAPK, 95°C for 5 min, 40 cycles at 95°C for 30 sec, 58°C for 30 sec, 72°C for 30 sec and a final extension of 5 min at 72°C. The MSP mixture contained 50 ng of bisulphite-treated DNA, 0.2 mM dNTPs, 2 mM MgCl_2_, 10 pmol of each primer, 1X PCR buffer and 1 unit Taq polymerase at a final volume of 50 *μ*l. The 10 *μ*l PCR products were then loaded onto 1.5% (m/v) agarose gel, electrophoresed and visualized under ultraviolet light subsequent to being stained with ethidium bromide.

### 5-Aza-deoxycytidine (DAC) treatment of the Tca8113 cell line

The Tca8113 cells were cultured in Iscove’s modified Dulbecco’s medium (Invitrogen Gibco) supplemented with 10% (v/v) newborn calf serum. The cells in the logarithmic proliferative phase were seeded in a 96-well plate at a density of 5×10^3^ cells/well and cultured in an incubator at 37°C with 5% CO_2_ saturated humidity for 24 h. The cells were then treated with 1, 3 or 5 *μ*M DAC (Sigma-Aldrich, St. Louis, MO, USA) for 72 h, with a fresh medium containing DAC that was replenished every 24 h. The cells in the treatment and control groups were harvested. Cell viability was detected using MTT and the cell morphology was observed under a contrast phase microscope. Apoptosis of the Tca8113 cells was studied using an Annexin V-PI dual staining assay and TUNEL. Demethylation of the Apaf-1 and DAPK promoter regions was detected by MSP and the mRNA expression of each gene was detected by RT-PCR.

### Statistical analysis

Quantitative data were expressed as arithmetic mean ± standard deviation (SD) and analyzed using the SPSS program (SPSS^®^ release 16.0, Chicago, IL, USA). mRNA expression in the normal oral mucosa and OSCC samples was analyzed by a t-test. The results were analyzed using the relative optical density of the target gene ratio to β-actin (mRNA index). mRNA expression was considered to be decreased in tumor tissues if the mRNA index was <50% than that in normal tissues and was considered normal if the mRNA index was ≥50% that in normal tissues. Pearson correlation was performed to assess Apaf-1 and DAPK gene expression in OSCC tissues. The association between mRNA expression and other clinical parameters, including the pathological grade, age and gender of patients, was studied using the χ^2^ test. Gene methylation was compared between the normal oral mucosa and OSCC groups using the χ^2^ test. The χ^2^ test was also performed to study the association between gene methylation and mRNA expression. A one-way analysis of variance was used to evaluate the statistical significance in the cell experiment. The P-value was evaluated according to Tukey’s method. All P-values were two-sided and P=0.05 was considered to indicate a statistically significant difference.

## Results

### RT-PCR

**Figure 1.**
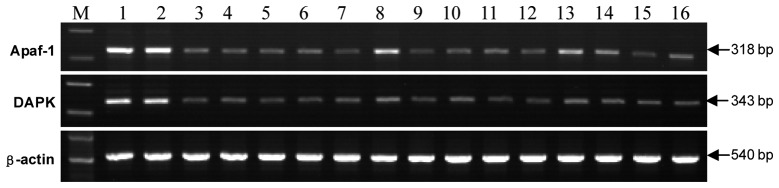
Representative examples of RT-PCR analyses of Apaf-1, DAPK and β-actin. Lanes 1 and 2 show samples of normal oral mucosa. Lanes 3–16 show samples of OSCC. RT-PCR, reverse transcription polymerase chain reaction; Apaf-1, apoptosis protease activating factor-1; DAPK, death-associated protein kinase; OSCC, oral squamous cell carcinoma; M, DL2000 Marker.

Apaf-1 and DAPK mRNA expression was detected in all 23 cases of normal oral mucosa and the positive rate was 100%, with no gene reduction or deletion. The mRNA index of the Apaf-1 and DAPK genes was 50% greater in two (3.77%) and three (5.66%) OSCC tissues compared to that of normal tissues, while in the remaining 51 (96.23%) and 50 (94.34%) cases, the mRNA index of the two genes showed a decreased expression (Fig. 1; Table III).

### MSP

**Figure 2.**
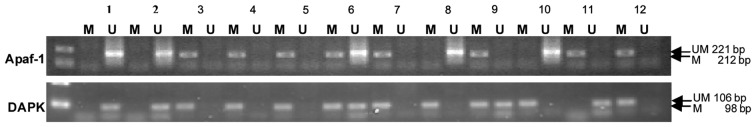
Representative examples of MSP analyses of Apaf-1 and DAPK. Lanes 1 and 2 show samples of normal oral mucosa. Lanes 3–12 show samples of OSCC. Lanes M show the amplified product with primers recognizing methylated sequences. Lanes U show the amplified product with primers recognizing unmethylated sequences. MSP, methylation-specific polymerase chain reaction (PCR); Apaf-1, apoptosis protease activating factor-1; DAPK, death-associated protein kinase; OSCC, oral squamous cell carcinoma; M, methylated; UM, unmethylated.

Methylation was not detected in the Apaf-1 and DAPK promoter regions in the normal oral mucosa samples. However, exhaustive methylation in the promoter region of the Apaf-1 gene was detected in 41 cases (77.36%) and partial methylation in 5 cases (9.43%) of OSCC samples. Exhaustive methylation in the promoter region of the DAPK gene was observed in 30 cases (56.60%) and partial methylation in 8 cases (15.09%) of OSCC samples. Apaf-1 and DAPK promoter methylation correlated with a decreased mRNA expression in the OSCC tissues (Fig. 2; Table IV).

### DAC treatment of the Tca8113 cell line

**Figure 3.**
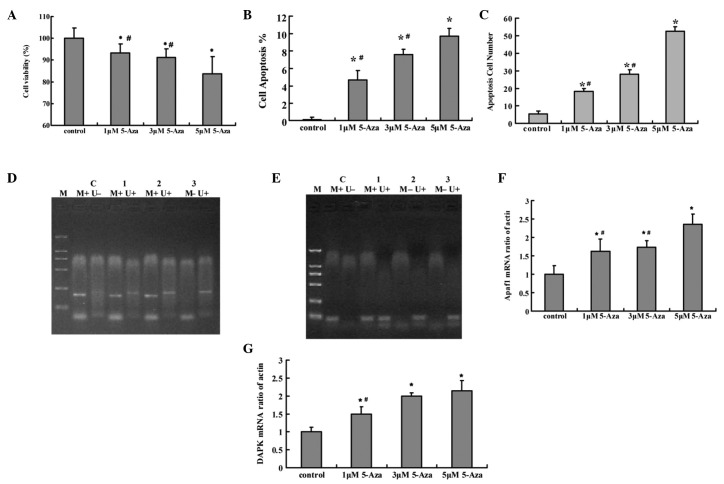
Analyses of Tca8113 tongue cells treated with DAC. (A) Cell viability analyses (MTT). (B) Cell apoptotic rate analyses (Annexin V-PI dual staining assay). (C) Cell apoptotic rate analyses (TUNEL). MSP analyses of (D) Apaf-1 and (F) DAPK. RT-PCR analyses of (F) Apaf-1 and (G) DAPK. DAC, 5-aza-2′-deoxycytidine; MSP, methylation-specific polymerase chain reaction (PCR); Apaf-1, apoptosis protease activating factor-1; DAPK, death-associated protein kinase; RT-PCR, reverse transcription-PCR.

Following treatment with DAC, the Tca8113 cell viability rate was significantly decreased compared with that of the control group (P<0.05). Apoptosis of the Tca8113 cells was induced by DAC and was also significantly different compared with that of the control group (P<0.05). A dose-dependent effect was observed in the cell viability and apoptosis. The two genes were demethylated following treatment with DAC and the most significant effect was observed in the 5 *μ*M DAC group. Apaf-1 and DAPK mRNA expression in the Tca8113 cells was significantly increased following treatment with DAC compared with the control group (P<0.05). A dose-dependent effect was observed in the mRNA expression. (Fig. 3)

### Statistical analysis

Apaf-1 and DAPK mRNA expression was significantly decreased in OSCC tissues compared with the normal oral mucosa samples (P<0.01). The Pearson correlation coefficient was 0.950 (P<0.01), which indicated that a correlation existed between the expression of the two genes in the OSCC tissues (Table V). The total methylation rate of the Apaf-1 and DAPK genes was 86.79% (46/53) and 71.69% (38/53), respectively in the OSCC tissues, which is significantly different compared with the normal oral mucosa samples (P<0.01). Apaf-1 and DAPK mRNA expression and promoter methylation were not shown to be significantly correlated with the pathological grade, age and gender of the patients (P>0.05; Tables VI and VII).

## Discussion

Epigenetics is a study of heritable changes that occur in gene function without changes in the sequence of nuclear DNA (9). The understanding of epigenetic mechanisms, including DNA methylation and changes in chromatin structure, has shown rapid progress (10). These changes may induce gene silencing, imprinting, paramutation and RNA interference. Therefore, abnormal modification may lead to tumorigenesis (11). Results of studies have demonstrated that Apaf-1 and DAPK are tumor suppressor genes, which rarely mutate. Therefore, the mechanism underlying the loss of function in the genes may be closely associated with promoter hyper-methylation (12–14).

The functional significance of Apaf-1 and DAPK methylation in OSCC is uncertain. Apaf-1 and DAPK mRNA expression and promoter hypermethylation status in OSCC have never been reported previously. The present study demonstrated that the expression of the Apaf-1 and DAPK mRNA indices was decreased (96.23 and 94.34%, respectively) and the gene promoter region was hypermethylated (86.79 and 71.69%, respectively) in OSCC. Further analysis showed that hypermethylation of Apaf-1 (P<0.05) and DAPK (P<0.01) promoter regions correlated with a decreased mRNA expression in the tumor tissues. The results indicate that epigenetic alterations of the Apaf-1 and DAPK genes exist and may play a role in OSCC.

As the core of the apoptosome complex, the Apaf-1 gene is a significant pro-apoptotic factor in the mitochondrial apoptosis pathway (15) and may be directly or indirectly associated with a number of diseases (16,17). Since the Apaf-1 gene is rarely mutated, promoter methylation appears to be more frequent in tumors. (18–20). As a positive regulator of apoptosis (21), the DAPK gene participates in numerous transduction pathways that are mediated by p53, TNF-α, Fas, TNF-β and other apoptotic factors, which also undergo promoter hyper-methylation in various types of tumors. An analysis of DNA hypermethylation in the serum of patients with non-small-cell lung carcinoma has shown that the DAPK hypermethylation frequency was 68.4% (22). DAPK is frequently methylated in malignant mesothelioma, which is associated with gene silencing and may be of prognostic significance (23). Studies have shown the DAPK gene to be hypermethylated in head and neck squamous cell carcinoma (24), bladder cancer (25), B-cell lymphoma (26) and cervical cancer (27).

In the present study, following statistical analysis, the expression and hypermethylation of Apaf-1 and DAPK was not significantly correlated with the pathological grade, age and gender of the patients (P>0.05). The Pearson correlation coefficient was 0.950 (P<0.01), indicating that the expression of the two genes in the OSCC tissues were correlated. Since they are the significant genes in the DAP kinase/p53/Apaf-1 apoptotic pathway, obtaining information in future studies may provide novel information for the treatment of OSCC.

The results have shown that Apaf-1 and DAPK promoter hypermethylation correlated with a decreased mRNA expression, indicating that the two genes may regain expression following demethylation. A dynamic pattern of methylation requires the presence of methylating and demethylating activities. The most controversial issue in the DNA methylation field is the question of whether DNA methylation is reversible (28). The DNA methylation reaction is catalyzed by DNA methyltransferase (DNMT). The consensus is that DNMT inhibitors are only active in replicating cells, through the passive inhibition of DNA methylation by blocking DNMT1 during DNA synthesis (29). As a type of DNMT1 inhibitor, DAC has been shown to reactivate tumor suppressor genes that have been silenced by promoter DNA methylation, in order to restore their function of inducing apoptosis, inhibiting tumor cell growth and providing therapeutics for chemotherapy-resistance tumors (30). Studies have indicated that DAC may be able to restore the function of the Apaf-1 gene in acute myeloid leukemia (31) and induce apoptosis of bladder cancer cells by reversing the unmethylated status of the DAPK promoter (32). The mechanism of function is associated with the induction of terminal differentiation, senescence or apoptosis, resulting in an irreversible loss of proliferative potential (33). Since Apaf-1 and DAPK promoter hypermethylation has been detected in OSCC, the present study aimed to confirm the demethylation role of DAC on the two genes in OSCC cells. The results revealed that apoptosis of the Tca8113 cells was induced by DAC. Furthermore, the cell viability rate was significantly decreased and the cell apoptotic rate was significantly increased in the Tca8113 cells that were treated by DAC. Demethylation of the Apaf-1 and DAPK genes correlated with the upregulation of the mRNA, indicating that methylation is responsible for the inactivation of Apaf-1 and DAPK in OSCC. A dose-dependent effect was observed in cell viability, apoptosis and mRNA expression. In the present study, the mRNA expression of Apaf-1 and DAPK was upregulated following gene demethylation and the genes were able to exert their pro-apoptotic roles.

In conclusion, the present study is consistent with numerous observations in carcinogenesis that have identified the loss of Apaf-1 and DAPK as a key feature in tumor progression. The occurrence and development of OSCC may be closely associated with a significant decrease in Apaf-1 and DAPK mRNA expression. In OSCC tissues, the Apaf-1 and DAPK promoter regions are usually methylated. Gene hypermethylation frequently leads to a decrease in Apaf-1 and DAPK mRNA expression. The Apaf-1 and DAPK gene expression was increased following demethylation, which promoted apoptosis of the tumor cells. Therefore, Apaf-1 and DAPK may serve as significant diagnostic markers or potential targets for OSCC treatment. Further research should clarify the DAPK/p53/Apaf-1 apoptosis pathway and its association with other pathways. Since 5-Aza-2′-deoxycytidine has limitations in clinical application, drug combination studies are also a significant aspect in additional investigations.

## Figures and Tables

**Table I.** Specific primers used in PCR.

Gene name	Primer sequence	Length (bp)	Product size (bp)
Apaf-1			
F	5′-TTG CTG CCC TTC TCC ATG AT-3′	20	318
R	5′-TCC CAA CTG AAA CCC AAT GC-3′	20	
DAPK			
F	5′-GAT AGA AAT GTC CCC AAA-3′	18	343
R	5′-TCT TCT TTG GAT CCT TGA-3′	18	
β-actin			
F	5′-GTG GGG CGC CCC AGG CAC CA-3′	20	540
R	5′-CTC CTT AAT GTC ACG CAC GAT TTC-′	24	

PCR, polymerase chain reaction; Apaf-1, apoptosis protease activation factor-1; DAPK, death-associated protein kinase, F, forward; R, reverse.

**Table II.** Specific primers used in MSP.

Gene name	Primer sequence	Length (bp)	Product size (bp)	Reference
Apaf-1				
Methylation				
F	5′-GAG GTG TCG TAG CGG TAT TC-3′	20	212	(3)
R	5′-CGA AAA TTA ACG AAA TAA ACG TC-3′	23		
Unmethylation				
F	5′-ATT TGA GGT GTT GTA GTG GTA TTT G-3′	25	221	
R	5′-ACC TCC AAA AAT TAA CAA AAT AAA CAT-3′	27		
DAPK				
Methylation				
F	5′-GGA TAG TCG GAT CGA GTT AAC GTC-3′	24	98	(8)
R	5′-CCC TCC CAA ACG CCG -3′	15		
Unmethylation				
F	5′-GGA GGA TAG TTG GAT TGA GTT AAT GTT-3′	27	106	
R	5′-CAA ATC CCT CCC AAA CAC CAA-3′	21		

MSP, methylation-specific polymerase chain reaction; Apaf-1, apoptosis protease activating factor-1, DAPK, death-associated protein kinase; F, forward; R, reverse.

**Table III.** Statistical analysis of Apaf-1 and DAPK mRNA expression.

Gene	Tumor (n=53)	Control (n=23)	P-value
Apaf-1/β-actin	0.11±0.02	0.52±0.02	P<0.01
DAPK/β-actin	0.10±0.03	0.80±0.01	P<0.01

Data are presented as mean ± standard deviation (SD). Apaf-1, apoptosis protease activating factor-1; DAPK, death-associated protein kinase.

**Table IV.** Association between the methylation status and mRNA expression downregulation of Apaf-1 and DAPK.

Gene	mRNA decrease (n)	mRNA normal (n)	χ^2^	P-value
Apaf-1				
Methylated	46	0	13.658	P<0.01
Unmethylated	5	2		
DAPK				
Methylated	38	0	8.256	P<0.01
Unmethylated	12	3		

Apaf-1, apoptosis protease activating factor-1; DAPK, death-associated protein kinase.

**Table V.** Pearson correlation analysis between Apaf-1 and DAPK.

Gene	n	Mean ± SD	P-value
Apaf-1	53	0.5113±0.2131	P<0.01
DAPK	53	0.2944±0.2922	

Apaf-1, apoptosis protease activating factor-1; DAPK, death-associated protein kinase; SD, standard deviation.

**Table VI.** Association between the mRNA expression of Apaf-1 and DAPK and pathological grade, age and gender of the patients.

Variable	n	Apaf-1 mRNA		χ^2^	P-value	DAPK mRNA		χ^2^	P-value
		Decreased	Normal			Decreased	Normal		
Grade, n				3.719	>0.05			1.512	>0.05
I	19	17	2			17	2		
II	25	25	0			24	1		
III	9	9	0			9	0		
Gender, n				0.019	>0.05			0.183	>0.05
Male	29	28	1			27	2		
Female	24	23	1			23	1		
Age (years), n				2.711	>0.05			0.701	>0.05
<55	23	21	2			21	2		
≥55	30	30	0			29	1		

Apaf-1, apoptosis protease activating factor-1; DAPK, death-associated protein kinase.

**Table VII.** Association between the Apaf-1 and DAPK methylation status and the pathological grade, age and gender of patients.

Variable	n	Apaf-1		χ^2^	P-value	DAPK		χ^2^	P-value
		+	−			+	−		
Grade				2.421	>0.05			0.266	>0.05
I	19	15	4			13	6		
II	25	22	3			18	7		>0.05
III	9	9	0			7	2		
Gender				0.458	>0.05			0.547	>0.05
Male	29	26	3			22	7		
Female	24	20	4			16	8		
Age (years)				0.620	>0.05			0.359	>0.05
<55	23	19	4			15	8		
≥55	30	27	3			23	7		

Apaf-1, apoptosis protease activating factor-1; DAPK, death-associated protein kinase;

+, methylated;

−, unmethylated.
